# From “satisfaction of search” to “subsequent search misses”: a review of multiple-target search errors across radiology and cognitive science

**DOI:** 10.1186/s41235-021-00318-w

**Published:** 2021-08-28

**Authors:** Stephen H. Adamo, Brian J. Gereke, Sarah Shomstein, Joseph Schmidt

**Affiliations:** 1 Department of Cognitive Psychology, University of Central Florida, Orlando, USA; 2 Department of Neuroscience, University of Texas at Austin, Austin, USA; 3 Department of Cognitive Neuroscience, The George Washington University, Washington, USA

**Keywords:** Subsequent search misses, Satisfaction of search, Visual search, Multiple-target search, Attentional template

## Abstract

For over 50 years, the satisfaction of search effect has been studied within the field of radiology. Defined as a decrease in detection rates for a subsequent target when an initial target is found within the image, these multiple target errors are known to underlie errors of omission (e.g., a radiologist is more likely to miss an abnormality if another abnormality is identified). More recently, they have also been found to underlie lab-based search errors in cognitive science experiments (e.g., an observer is more likely to miss a target ‘T’ if a different target ‘T’ was detected). This phenomenon was renamed the subsequent search miss (SSM) effect in cognitive science. Here we review the SSM literature in both radiology and cognitive science and discuss: (1) the current SSM theories (i.e., satisfaction, perceptual set, and resource depletion theories), (2) the eye movement errors that underlie the SSM effect, (3) the existing efforts tested to alleviate SSM errors, and (4) the evolution of methodologies and analyses used when calculating the SSM effect. Finally, we present the attentional template theory, a novel mechanistic explanation for SSM errors, which ties together our current understanding of SSM errors and the attentional template literature.

## Introduction

Visual search is defined as the process of looking for targets amongst distractors and is a key cognitive ability we use to navigate the visual world. Visual search can be as mundane as looking for a car in a crowded parking lot or as critical as searching a chest image for signs of cancer. Much is known about attention and object processing by studying visual search. For example, the detection of the target may be easier or harder depending on how similar the perceptual features (e.g., color and shape) are between targets and distractors (e.g., Duncan & Humphreys, 1989). Target detection is also influenced by the prevalence of targets (e.g., Mitroff & Biggs, 2014; Wolfe et al., 1997) and the amount of visual information (e.g., clutter) presented within the visual search image (e.g., Rosenholtz et al., 2007). Finally, much is known about how a target’s location is selected within the search environment (e.g., Olivers et al., 2011; Treisman, 1991; Wolfe, 1994).

While single-target search is well-researched (see Eckstein, 2011; Nakayama & Martini, 2011 for a review), much less is known about multiple-target search in which more than one target may be present within a search image. Searching for multiple targets can result in a miss of one target when a different target is detected in the image. Originally known as the satisfaction of search (SOS) effect in radiology (Smith, 1967), and recently renamed the subsequent search miss (SSM) effect in cognitive science (Adamo et al., 2013), the field of radiology has studied this phenomenon for over 50 years. (Smith, 1967; Tuddenham, 1962). It is especially problematic in radiology because it is important to detect all abnormalities within an image (e.g., missing a cancer within an image can be life-threatening). SSM errors can account for up to one-third of certain radiological reading errors (Anbari & West, 1997) and are found to occur in a wide variety of radiological exams including abdominal radiography, skeletal radiography, chest radiography, and multiple-trauma patient scans (e.g., Ashman et al., 2000; Berbaum et al., 1994; Berbaum et al., 1996; Berbaum et al., 1998; Berbaum et al., 2015; Franken et al., 1994; Samuel et al., 1995).

Cognitive science began investigating SSM errors approximately 10 years ago and demonstrated that they were a general cognitive phenomenon. Novice observers (e.g., university undergraduates) also commit SSM errors in simplified-search displays (e.g., searching for target ‘T’s amongst distractor ‘L’s; Fleck et al., 2010). This research demonstrated that SSM errors were likely caused by a universal limitation in how observers search rather than something unique to radiological images or radiologists as diagnosticians. As such, cognitive scientists have joined medical images perception researchers and radiologists in an effort to understand why SSM errors occur, with the ultimate goal of learning how to reduce them.

In subsequent sections, this review will discuss current SSM theories and the current state of the literature in both radiology and cognitive science. Eye-tracking, both in terms of its utility to understand SSM errors and the attempts that have been made within radiology to reduce SSM errors, will be discussed. The various methods and analyses that have been used to measure and calculate SSM errors will also be defined and reviewed. Finally, we will present a new SSM theory and discuss potential avenues that SSM research should take.

## Current SSM error theories

Three distinct theories were initially proposed within radiology to explain why SSM errors occur. The Satisfaction theory hypothesized that SSM errors are a "temporal" problem—observers make SSM errors because they become “satisfied” with the meaning of an image (i.e., diagnoses) after finding a first abnormality, which causes them to prematurely terminate their search (Smith, 1967; Tuddenham, 1962). The Perceptual Set theory hypothesized that SSM errors were a “perceptual” problem—observers are more likely to search for a target that is similar to the target they identified first, thereby making them more likely to miss a dissimilar, second target (Berbaum et al., 1990, 1991). The Resource Depletion theory hypothesized that SSM errors are a "cognitive resource" problem—the process of detecting the first target consumes cognitive resources (e.g., attention and working memory), which subsequently leaves fewer resources available to identify an additional target (Berbaum et al., 1991; Cain & Mitroff, 2013). Individually these theories have all received empirical support, suggesting that there is a temporal, perceptual, and cognitive resource component to SSM errors (Adamo et al., 2013, 2015, 2017, 2018; Biggs et al., 2015; Cain & Mitroff, 2013; Cain et al., 2013; Gorbunova, 2017; Samuel et al., 1995; Stothart & Brockmole, 2019; Stothart et al., 2018). However, individually no theory can account for all of the SSM error-related effects. Below, we will discuss the research from radiology and cognitive science that was used to investigate each SSM theory (see Table 1 for a short description of the research investigating each theory).

**Table 1:** Summary of SSM Theories and Their Respective Research

**Current SSM theories**	**Research**
**Satisfaction**	**Do not support**
Observers become satisfied with the meaning of an image after finding the first target and prematurely terminate search. (Smith, 1967; Tuddenham, 1962)	Berbaum et al. (1991)-Total time spent searching for targets is not significantly different in single-and multiple-target images
	Berbaum et al. (1998)-Observers have similar gaze-dwell times in “native” target area on single and multiple-target images
	Fleck et al. (2010)-Total time spent searching for targets is similar in single-and multiple-target search displays (“Appendix”)
	Cain et al. (2013)-Observers rarely terminate search immediately after detecting the first target
	**Support**
	Samuel et al. (1995)-Less total time spent searching in multiple-target compared to single-target images
	Adamo et al. (2018)-Observers who spend less time searching after first target detection are more likely to commit an SSM error
	Stothart and Brockmole (2019)-Observers who are less likely to “expect” a second target are more likely to commit an SSM error
**Perceptual set**	**Do not support**
Observers are biased to search for targets similar to a detected target and are more likely to miss dissimilar targets (Berbaum et al., 1990, 1991)	Fleck et al. (2010)-Observers commit SSM errors for targets that are similar and dissimilar in salience
	**Support**
	Berbaum et al. (2001)-Reduced SSM effect when abnormalities were similar in severity (i.e., both abnormalities were minor compared to a major and minor abnormality)
	Mitroff et al. (2015)-Reduced SSM effect when targets were identical compared to when they were not identical
	Biggs et al. (2015)-Reduced SSM effect when targets were perceptually and categorically similar compared to when they were dissimilar
	Gorbunova (2017)-Improved second-target detection when a first and second target have more perceptual features in common
**Resource depletion**	**Support**
A detected first target consumes attentional and working memory resources leaving fewer resources readily available to detect an additional target (Berbaum et al., 1991; Cain & Mitroff, 2013)	Adamo et al. (2013)-An “attentional blink” can cause SSM errors
	Cain and Mitroff (2013)-Reduced working memory load, by removing or changing the first target after detection, reduces SSM errors
	Adamo et al. (2015)-Clutter around a second target increases SSM errors
	Adamo et al. (2017)-Individual differences in attentional modulation (i.e., the width of their attentional blink) and vigilance correlate with the SSM effect
	Stothart et al. (2018)-Movement of targets and distractors increases the SSM effect

### Satisfaction theory

In the 1960s SSM errors were first noted in the radiology literature. Researchers at the time believed that search errors were caused by a feeling of "satisfaction." After finding a first abnormality (i.e., target), it was predicted that radiologists became “satisfied” with the meaning of the image and prematurely terminate their search, which caused them to miss the remaining abnormality (Smith, 1967; Tuddenham, 1962). While this theory is the source of the original name “satisfaction of search,” it was not empirically tested until about 30 years after it was proposed. The Satisfaction theory was first tested by comparing the total time radiologists spent searching single-abnormality and multiple-abnormality plain-film chest radiographs (Berbaum et al., 1991). The researchers sought to determine if less time was spent searching in multiple-abnormalities images compared to single-abnormality images. Presumably, since finding one of two abnormalities should be faster than finding one of one abnormality (i.e., you have double the chances of finding an abnormality), termination of search was predicted to be faster in multiple abnormality images if observers were “satisfied.” Initially, it appeared that radiologists were *not* “satisfied” after a first abnormality was detected, but they instead spent the same amount of time searching through single- and multiple-abnormality images. In other words, radiologists *did not* prematurely terminate their search after finding the first abnormality but instead continued to search for additional abnormalities. To further investigate "satisfaction", Samuel et al. (1995) found that when radiologists were given up to 30 seconds for reading chest radiographs, radiologists *did* spend less time searching multiple-abnormality images compared to single-abnormality images. However, these results are difficult to interpret because radiology participants ran out of time on more than 80% of trials. Thus, it is unclear if these results would persist if radiologists were given more time to search.

Various approaches have been employed in cognitive science to test the Satisfaction theory (Adamo et al., 2018; Cain et al., 2013; Stothart & Brockmole, 2019). For example, Cain et al. (2013) used eye-tracking to determine if observers fixated additional items after finding the first target. They found that observers continued to fixate search items after finding the first target, suggesting observers were not “satisfied.” Later the same researchers found support for the Satisfaction theory by using an individual differences approach (Adamo et al., 2018). There was a correlation between the time individuals spend searching after finding a first target and the likelihood of committing an SSM error. If observers searched for less time after finding the first target, then they were more likely to miss an additional target. As a control, a separate vigilance task (i.e., to measure sustained attention over time) was conducted. Time spent searching *after* finding a first target was still predictive of SSM errors even when accounting for how vigilant observers were. This finding suggests that above and beyond how attentive observers were, those who were more “satisfied” produced the most SSM errors. As an explanation for individual differences in time spent searching after finding a first target, target “expectancy” (i.e., do observers expect an additional target) was proposed (Stothart & Brockmole, 2019). After observers found the first target, they were sometimes primed with the question of whether or not they expected to find a second target. Their “expectancy” biases derived from these trials were positively correlated with how long they spent searching after finding the first target. If observers expected another target, they were more likely to search for longer after finding the first target.

### Perceptual set theory

The Perceptual Set theory proposes that once observers find a target, they are primed to find similar targets and less likely to find dissimilar targets (Berbaum et al., 1990, 1991). Radiology has garnered suggestive evidence for the validity of perceptual set theory (Berbaum et al., 2001). In a study reviewing multiple-trauma radiographs of obvious versus subtle fractures, there was a reduced SSM effect when both fractures were minor compared to when one was major and the other was minor (Berbaum et al., 2001). The perceptual set theory suggests that finding the more obvious fracture first primed radiologists to search for additional obvious, similar fractures, making them more likely to miss subtle injuries. Alternatively, this could also be seen as evidence for the Resource Depletion theory—presumably it takes more cognitive resources to detect a subtle abnormality compared to an obvious one. Therefore, radiologists were more likely to find two obvious abnormalities compared to an obvious and subtle abnormality. One shortcoming of this study is that it did not investigate the perceptual similarity of the targets (i.e., whether targets shared similar visual features or not) making it hard to determine whether radiologists were more likely to find perceptually similar abnormalities.

To test how the perceptual similarity between targets affects detection, cognitive scientists have utilized simplified-search displays where target similarity can be easily manipulated by changing perceptual features, such as a target’s color and shape (e.g., Biggs et al., 2015; Fleck et al., 2010; Gorbunova, 2017). The first SSM paper in cognitive science reproduced the result from radiology by demonstrating that observers were more likely to miss a low-salience target (i.e., a less obvious target) after finding a high-salience target (i.e., a more obvious target; Fleck et al., 2010). However, in the same study, an SSM effect was also found when observers searched for multiple targets that were of low salience. This finding suggests that the Perceptual Set theory *cannot* be the sole explanation of SSM errors because SSM errors still occurred when targets were highly similar in salience. Stronger evidence for the Perceptual Set theory was found using a wide array of targets in a simulated baggage screening task (Mitroff et al., 2015; Biggs et al., 2015). Unlike previous SSM studies that had a small number of possible target types, Biggs et al. (2015) examined similarity in close to 100 possible targets across three conditions: (1) whether the two targets were identical (e.g., two pistols), (2) whether two targets were the same color, and (3) whether two targets were from the same category (e.g., the targets were guns or gun related items such as a gun and bullets). While SSM errors persisted in all three similarity conditions, there were more SSM errors when the targets were dissimilar compared to when they were similar. Furthermore, the biggest decrease in SSM errors occurred when both targets were identical. These findings suggest that while SSM errors occur when there are multiple-target types, they are reduced when targets are perceptually and conceptually similar. However, given that SSM errors still occur no matter how similar targets are (i.e., even if targets are identical; Mitroff et al., 2015), this finding also suggests that the Perceptual Set theory alone cannot explain all SSM errors.

### Resource depletion theory

According to the original resource depletion theory, SSM errors were predicted to occur because a found target captures attention, which expends the attentional resources readily available to process an additional target (Berbaum et al., 1991). While this theory has not been explicitly tested in radiology, cognitive science examined whether visual working memory resources allocated to a first target leaves fewer resources readily available for additional target processing (Cain & Mitroff, 2013). Visual working memory has been demonstrated to store both the featural and spatial aspects of items within a visual search display (e.g., Beck et al., 2006; Hollingworth et al., 2001; Körner & Gilchrist, 2008; Peterson et al., 2001; Takeda, 2004; Woodman & Chun, 2006). Cain and Mitroff (2013) found that when a first target was removed from a search display after being detected, there was a decrease in SSM errors relative to when the first target remained in the search display. They suggested that the removal of the first target reduced working memory load. With a reduced working memory load, working memory resources could be re-allocated to detect a second target, which results in improved second-target detection. Cain et al., (2014) then found that if search ended immediately after finding the first target in a dual-target display, and the same display was later repeated a few displays later without the first target, second-target detection improved. There was no difference in accuracy for single-target displays and accuracy for a “second-target” when the first target was removed and the display was repeated with only the remaining target. Together these results suggest that visual working memory plays a role in SSM errors and observers' working memory resources are allocated to a first target after detection.

The Resource Depletion theory has additional support outside of the scope of visual working memory (Adamo et al., 2013, 2015, 2017; Stothart et al., 2018). Adamo et al. (2013)^1^ provided some of the first evidence linking attention to SSM errors by demonstrating that a well-studied cognitive phenomenon may underlie SSM errors. The paper demonstrated that a self-induced attentional blink could cause SSM errors when a second target is fixated shortly after a first target’s fixation (~ 200–500 ms after a first target was fixated). An attentional blink is defined as a decrease in second-target identification in a temporal visual search (e.g., Raymond et al., 1992). An attentional blink has been shown to occur when a second target appears ~ 200–500 ms after a first target (e.g., Broadbent & Broadbent, 1987; Chun & Potter, 1995). While attentional blinks are traditionally examined in rapid serial visual presentation streams (e.g., where search items are presented serially for a brief duration in the same location), Adamo et al. (2013) found that they can occur in a spatial visual search as well. By finding that a subset of SSM errors can be directly attributed to the attentional blink suggests that SSM errors can be attributed to the attentional processing of a first target. Alternatively, this could also explain why observers are more likely to miss a second target when they prematurely terminate search after finding a first target (i.e., the support for the Satisfaction theory; Adamo et al., 2018; Stothart & Brockmole, 2019). If observers terminate search after fixating a second target during the attentional blink window, they would be more likely to miss it compared to if the second target was fixated outside of the attentional blink window. More recent evidence also corroborated that an attentional blink was related to SSM errors (Adamo et al., 2017). Observers’ performance in an attentional blink task and a vigilance task both correlated with how often observers missed the second target after finding the first target. Observers with longer attentional blinks and observers with poorer vigilance (i.e., sustained attention over time) made more SSM errors compared to observers with shorter attentional blinks and better vigilance.

Other research related to attention and SSM errors demonstrated that clutter (i.e., distractors in close spatial proximity to a target; Adamo et al., 2015) and motion in a search display (Stothart et al., 2019) increased SSM errors. While clutter around a single target did not affect target detection, clutter around a second target (after a first target was found) reduced second-target detection (Adamo et al., 2015). This finding suggests that when the attentional processing of a first target is coupled with the increased attention needed to process the second target in clutter, observers are more likely to miss a second target. Similarly, when the distracting effects of motion within a search display (i.e., all search items could move) was coupled with the attentional processing of a first target, observers were more likely to make an SSM error compared to when the search display was stationary (Stothart et al., 2018).

Overall these findings suggest that when attention is allocated to first target processing, observers are more likely to miss a second target when: (1) attention has to be rapidly reallocated to process an additional target (i.e., an attentional blink), (2) more attention is needed to process a second target (e.g., when the second target is in a high clutter region), and (3) when less attention is available (i.e., when observers are less vigilant). These findings collectively provide strong support for the Resource Depletion theory. Unfortunately, there is currently no specific, theorized mechanism in the Resource Depletion theory that explains why cognitive resources are utilized after a first target is found and why there are fewer resources readily available to process a second target. Furthermore, there are no specific predictions made by the Resource Depletion theory to explain other sources of SSM errors such as those predicted by the Perceptual set theory (e.g., Biggs et al., 2015) and the Satisfaction theory (e.g., Adamo et al., 2018; Stothart & Brockmole, 2019). In the *Future Directions* section, we suggest a new SSM error theory that provides a mechanistic explanation for SSM errors and links the currently disparate SSM error findings and theories together.

## Eye movements and SSM errors

Converging evidence from radiology and cognitive science eye-tracking studies offer insight into the current SSM theories. Previous work in radiology utilized eye-tracking to categorize visual search misses into three distinct types of errors: sampling errors (also known as search errors), perceptual recognition errors, and decision-making errors (e.g., Nodine & Kundel, 1987). Below each type of error is presented and discussed in regards to SSM error theories.

### Sampling errors

Sampling errors are categorized as a situation when a target is never fixated (e.g., Nodine & Kundel, 1987). They do not appear to be a significant contributor when the abnormality of interest (i.e., the abnormality where the SSM effect was measured) was missed in chest and abdominal images (e.g., Berbaum et al., 1996, 2001; Samuel et al., 1995). However, in simplified search displays, sampling errors contributed to around 50% of SSM errors (Cain et al., 2013). While empirical evidence to explain this discrepancy is lacking, it may be attributed to differences in the search displays between the two fields and/or the expertise of the searchers. For example, in radiological images, radiologists know where an abnormality is more likely to appear, whereas in simplified search displays, the target locations are randomized and observers do not expect targets to appear in one spatial location of the display more so than another.

Sampling errors may speak to the Satisfaction account in that observers are less likely to fixate a second target if they terminate search quickly after finding a first target. While only a small proportion of SSM errors could be attributed to terminating search immediately after detecting a first target (Cain et al., 2013), sampling errors after detecting the first target have not been investigated. Sampling errors may also support the Resource Depletion account because depletion of cognitive resources may cause observers to dismiss the area where the target is located. There is a diminished ability to take up information in peripheral vision when working memory resources are consumed by a found target (Chan & Courtney, 1995; Takeda, 2004). These findings suggest that once a target is found in a multiple-target search, there could be a narrowing of the perceptual span (i.e., area in which information can be encoded during a fixation). This could increase the likelihood of an SSM error due to reduced guidance towards an additional target, making it less likely to be fixated and subsequently detected. Sampling errors could also support the Perceptual set account—if two targets are dissimilar, then a second target may not attract attention, and therefore, may not be fixated. It is unclear whether this actually occurs because target similarity has not been systematically manipulated in an SSM study while eye movements were tracked.

### Perceptual recognition and decision making errors

According to Nodine and Kundel (1987), perceptual recognition errors are defined as situations in which a target is fixated but not for a duration long enough for recognition. In contrast, decision-making errors are described as a fixation/cluster of fixations that is long enough to identify a target but is rejected by the observer as a target. A radiologist may fixate a cancer but ultimately decide it is a benign lesion for a myriad of reasons.

Errors when an abnormality is fixated but not identified account for the majority of SSM errors in radiology (e.g., Berbaum et al., 1996, 2000, 2001 Samuel et al., 1995). For example, Samuel et al. (1995) found that while about 90% of missed targets were fixated, there was a 20% increase in perceptual recognition errors in multiple-target searches compared to single-target searches. Furthermore, SSM errors can be exclusively perceptual recognition and decision-making errors if the first target is of great clinical significance (e.g., a major fracture in a radiograph; Berbaum et al., 2001).

Importantly, the distinction between perceptual and decision-making errors is determined by a single dwell time cut-off (i.e., how long observers fixate a given area), and therefore may be incorrect, given that different abnormalities require different dwell times for recognition. In an attempt to dissociate between perceptual recognition or decision-making SSM errors, without a dwell time metric, Berbaum et al. (2000) had radiologists verbally report their search techniques while reading chest radiographs. Verbal reports allowed researchers to determine if radiologists were “consciously” deciding whether an abnormality existed or not. With this method, the proportion of decision-making errors were reduced compared to a single dwell-time measure, suggesting that SSM errors were more likely to result from perceptual recognition errors (i.e., fixated but not recognized as an abnormality). Surprisingly, SSM errors were reduced with a verbal report, suggesting that radiologists may have been more deliberate and/or adopted a specific search strategy that they otherwise would not adopt in the non-verbal condition.

In cognitive science, around 50% of SSM errors could be attributed to perceptual recognition/decision-making errors in simplified search displays (observers fixated a second target but did not detect it; Cain et al., 2013). This proportion is less than those reported in radiology and may be driven by the types of search images used. In radiological images, a target abnormality can be difficult to distinguish from normal anatomy, which is why radiologists undergo intensive training to detect and assess abnormalities. In contrast, the search displays used in cognitive science SSM tasks typically have targets and distractors that are easily distinguishable (i.e., targets require very little training to identify and targets and distractors typically do not overlap). Therefore, when a target is fixated within a simplified display, it may be more likely recognized as a target compared to abnormalities in radiological images.

These types of errors broadly support the Resource Depletion account; a first target utilizes valuable cognitive resources leaving fewer resources readily available to process an additional target. These types of errors do not support the Satisfaction theory given that observers still searched for additional targets and failed to recognize them. Perceptual recognition and decision-making errors could support the Perceptual set theory in that observers would be more likely to recognize a perceptually similar target after detecting the first target. However, it is unclear whether they support the Perceptual Set theory because target similarity has not been systematically manipulated while eye movements are monitored.

## Intervention attempts to alleviate SSM errors

When SSM errors were first discovered, Tuddenham (1962) proposed to have radiologists search more systematically (e.g., going from left to right within the film) as opposed to freely searching the images to alleviate SSM Errors. He also proposed that radiologists could use an automatic scanning device during training so that radiologists learn how to systematically search. To determine whether systematic searching could actually improve SSM errors, researchers had radiologists use either a checklist (Berbaum et al., 2006) or a vocalized checklist (Berbaum et al., 2016) when searching. While checklists can be beneficial in hybrid single-target searches, where observers search for categorically defined and specific targets (Nartker et al., 2020), they did affect SSM error rates. These findings suggest that the possibility of multiple targets within an image may affect the efficacy of checklists.

Radiologists have also tried using target detection tools, such as contrast-enhanced imaging (Franken et al., 1994) and computer-aided detection (Berbaum et al., 2007) to reduce SSM errors. In comparison to plain film radiographs, contrast-enhanced images are created with intravenously injected dyes to better delineate areas with possible abnormalities. Unfortunately, contrast-enhanced images were found to actually *increase* SSM errors relative to plain-film radiographs (Franken, et al., 1994). The authors speculated that more SSM errors occurred because attention was drawn to the high-contrast areas, leaving lower-contrast areas containing abnormalities unexplored. Similar to contrast-enhanced imaging, the purpose of computer-aided detection (CAD) is to use computer algorithms to mark suspicious areas of an image. Unfortunately, researchers found no difference in SSM errors between CAD-marked images and plain radiographs (Berbaum et al., 2007; but see Schartz et al., 2013). Although these findings are disappointing, they emphasize the importance of understanding the mechanisms underlying SSM errors.

Limited research within cognitive science focuses on interventional strategies for SSM errors. The key finding from cognitive science is that SSM errors are reduced if a first target is removed after detection, whether at the moment of detection (Cain & Mitroff, 2013) or by repeating the display at a later time without the first target (Cain et al., 2014). Beyond this research, more image-specific intervention research (e.g., blocking out or removing potential abnormalities from view after they have been marked as a potential point of interest) or observer-intervention research (e.g., motivation or training) is needed to determine potential new ways that SSM errors might be mitigated.

## SSM methods and calculations

### Radiology

When the study of SSM errors resurfaced in the 1990s, Berbaum et al. (1990) set the standard for how SSM errors should be investigated and calculated in radiology. To determine if there was an SSM effect, researchers created identical images to compare performance for a specific target. One image would contain a native lesion (i.e., “test” target) while the other image would be identical except with the addition of a simulated pulmonary nodule (i.e., an “added” target). Nodules were chosen because they were one of the few abnormalities that could be simulated to look realistic in x-rays (and eventually in computed radiography) within chest images (Berbaum et al., 2016; Samei et al., 1997). Observers made binary decisions whether an abnormality was present via a mouse click and reported confidence levels in their decision. Search performance for the “test” target was then compared between the two images using signal detection measures such as receiver operating characteristic (ROC) curve analyses and measures such as A’ and d’ (see Berbaum et al., 2010a for a review). These measures allowed radiologists to take into account hit and false alarm rates when comparing single and multiple abnormality images. An SSM effect was thought to be present if search performance was worse for the “test” target with the “added'' target relative to when the “test” target was by itself. Importantly, except for the added nodule, these images were identical which helped to avoid any inadvertent confounds (e.g., more distracting clutter in a dual-abnormality image compared to a single-abnormality image). However, this design was not dependent upon the order of detection despite the proposed SSM theories assuming a temporal effect (i.e., how the detection of a first abnormality impacted a second abnormality’s detection) and many radiological studies used this same reproducible approach when investigating the SSM effect (e.g., Berbaum et al., 1991, 1994, 1996; Samuel et al., 1995). Sometimes identical images were created with just the “added” target so that an SSM effect could be assessed for the “added” target (e.g., Samuel et al., 1995) but rarely were non-identical images used for evaluation (Ashman et al., 2000).

SSM errors were eventually categorized for radiological images as either Type I or Type II (Berbaum et al., 2015). Type I SSM errors were characterized as a change in the ROC curve driven by a decrease in hit rate, with no change in false alarms. Type II SSM errors were characterized as a conservative change in the ROC curve driven by a decrease in hit rate with a reduction in false alarms (i.e., observers were overall more reluctant to report any abnormality was present). When SSM errors from early studies that used plain film chest radiographs (Berbaum et al., 1990, 2000) were reanalyzed, researchers found that there were primarily Type 1 SSM errors. In contrast, later studies that used computed chest radiographs were more likely to result in Type II SSM errors (Berbaum et al., 2015; Krupinski et al., 2017). It is unclear why the shift from Type I to Type II occured, but it did not appear to be driven by a change in the time observers spend viewing radiological images (Krupinski et al., 2017).

### Cognitive science

As the study of SSM errors transitioned to cognitive science (e.g., Cain et al., 2011; Fleck et al., 2010), researchers began using simplified search displays and different analyses to calculate SSM errors. The first SSM error study used randomized displays rather than identical ones for single- and multiple-target searches (Fleck et al., 2010). Similar to radiology, subjects made binary decisions with mouse clicks but no confidence ratings were recorded, which precluded the calculation of ROC curves. Instead, the SSM effect was calculated using only target hit rates.

**Fig. 1:**
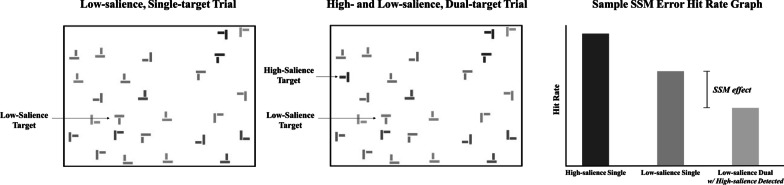
**Sample Single-target Display, Dual-target Display, and Hit Rate Graph for an SSM task:** In a standard cognitive science SSM task, observers are asked to search in simplified-search displays for ‘T’ shaped targets (where the crossbars of the two perpendicular rectangles perfectly bisect one another) amongst pseudo ‘L’ shaped distractors (two perpendicular rectangles do not perfectly bisect one another). Search items are typically randomized for rotation (e.g., 0, 90, 180, or 270 from a canonical ‘T’ shape) and location within the search display. Search items may also vary in “salience” (e.g., a darker gray or light gray color amongst a white background), which allows researchers to compare the hit rate of a specific type of target (e.g., low-salience ‘T’) between single and dual-target displays to calculate the SSM effect. The graph above depicts a typical SSM effect with a lower hit rate for a low-salience ‘T’ when it appeared in a dual-target display compared to when a low-salience ‘T’ appeared in a single-target display. These hypothetical results suggest that the detection of the low-salience target ‘T’ was affected by the detection of the high-salience target ‘T’ compared to when it was by itself in a search display (e.g., Fleck et al., 2010)

The most common cognitive science SSM paradigm utilized targets of different saliences—a low-salience target ‘T’ of a lighter gray color and a high-salience target ‘T’ of a darker gray color that could appear amongst predominantly low-salience distractor pseudo ‘L’s (see Fig. 1). The hit rate of the low-salience target on single-target trials was compared to the hit rate for a low-salience target when it appeared with a high-salience target on dual-target trials. The earliest way of calculating SSM errors was to compare the hit rate for a low-salience target when a high-salience target was detected on dual-target trials to the hit rate of a low-salience target on single-target trials. (Fleck et al., 2010; Cain et al., 2012).

The SSM calculation later changed to include the caveat that the high-salience target *had to be detected first* on the dual-target trials (e.g., Cain & Mitroff, 2013). The goal was to determine if the detection of a “first” high-salience target subsequently impacted the detection of a “second” low-salience target (compared to when the low-salience target appeared by itself). Given that all SSM theories to date were predicated on the detection of one target impacting another, this change was implemented to quantify the “subsequent” aspect of SSM errors. Additionally, this allowed for a direct comparison to the attentional blink literature, which is determined by how long/how many distractors appear between a detected first target and a second target (Adamo et al., 2013). When salience was manipulated in the experiment (i.e., See Fig. 1), this change resulted in removing dual-target trials, where a low-salience target was detected first, from the SSM error analysis (e.g., Adamo et al., 2013; Cain et al., 2013).

While the comparison of single-target hit rate to "second"-target hit rates was largely adopted by SSM error researchers (Adamo et al., 2015, 2017; Cheng & Rich, 2019; Gorbunova, 2017; Stothart & Brockmole, 2019; Stothart et al., 2018), it was soon discovered that this approach was inherently biased and could exaggerate the SSM effect (Adamo et al., 2019). Restricting the analyses to the “second” target on dual-target trials (and filtering out instances in which the low-salience target was detected first in cases where salience was manipulated), resulted in an inflated SSM effect. On dual-target trials, participants are more likely to find the “easier” of two targets first, resulting in an influence of “difficult-to-detect” target configurations composing the second-target hit rate measure. In the case of a salience manipulation, the easier trials in which the low-salience target was detected first were removed from the analysis. Therefore, when the second-target hit rate is compared to the single-target hit rate (which is composed of both “easy” and “difficult” configurations), the SSM effect was inflated.

Adamo et al. (2019) proposed a methodological correction by using a matched set of “triplet” stimuli, where each dual-target trial is balanced by two matching single-target trials. For these “matched” single-target trials, all search items were identical to those in the dual-target trial with one of the two targets replaced by a random distractor. This setup corresponds to the radiological methods originally suggested by Berbaum et al. (1990) and attempts to correct the bias in the SSM error calculation due to differences in target detection difficulties. The calculation correction compared the hit rate for "second" targets (i.e., targets that were not found first) to the hit rate for the same targets in matched single-target displays. This is a subtle difference from the method proposed by Berbaum et al., (1990) in which “test” target performance was assessed irrespective of order effects. While this new method was shown to reduce the estimated size of the SSM effect compared to previous methods, this more conservative approach did not eliminate the SSM effect.

More recently Becker et al. (2020) argued the above approach is limited by its restrictive experimental design (i.e., that using matched displays can limit the number of dual-target trials within an experiment as there has to be two matched single-target trials). Furthermore, they hypothesized that stimulus matching is an imperfect correction for biases caused by differences in second-target detection difficulty and argued that biases could exist beyond those induced by stimulus display properties. In general, any comparison of hit rates between dual- and single-target trials conflates potentially distinct contributions from either the addition or detection, of a second target. They proposed an alternative approach that does not rely on comparing second-target hit rate to single-target hit rate. Rather, they derived an unbiased expectation solely from dual-target trials to assess the probability of detecting a "test" target (i.e., the target you want to determine whether there is an SSM effect for) given that an "added" target (i.e., the target that can cause an SSM effect) is detected first. Specifically, they derived the equation:

1 $$\begin{aligned} P(A|B\;1{\text{st}})\, {:=}\, & \frac{{{\text{Dual trials}}\;w/B\;{\text{detected then}}\;A\;{\text{detected}}}}{{{\text{Dual trials}}\;w/B\;{\text{detected then}}\;A\;{\text{detected}} + {\text{dual trials}}\;w/{\text{only}}\;B\;{\text{detected}}}} \\ & \frac{ < }{ > }\frac{{P\left( A \right)P\left( B \right)P(A\; 2{\text{nd}} |A, B)}}{{P\left( A \right)P\left( B \right)P(A\; 2{\text{nd}} | A, B) + P\left( B \right)\left( {1 - P\left( A \right)} \right)}} \\ \end{aligned}$$

1.1 $$P\left( A \right)\, {:=}\, \frac{{{\text{Dual trials}}\;w/A\;{\text{detected}}}}{{\text{Dual trials}}}$$

1.2 $$P\left( B \right)\, {:=}\, \frac{{{\text{Dual trials}}\;w/B\;{\text{detected}}}}{{\text{Dual trials}}}$$

1.3 $$P(A\;2{\text{nd}}|A,B)\, {:=}\, \frac{{{\text{Dual trials}}\;w/B\;{\text{detected}}\;{\mathbf{then}}\;A\;{\text{detected}}}}{{{\text{Dual trials}}\;w/B\;{\text{detected}}\;{\mathbf{and}}\;A\;{\text{detected}}}}$$

The leftmost and middle parts of Eq. 1 describe how the *observed* conditional hit rate for the “test” target given that the “added” target was detected first (i.e., *P*(*A* | *B* 1st)) is calculated from the data (i.e., second-target hit rate for the “test” target). Instead of comparing this value to single-target hit rate, as was done in previous SSM error calculations (e.g., Cain & Mitroff, 2013; Fleck et al., 2010), the rightmost part of Eq. 1 describes how the theoretically derived *expected* value (i.e., the value expected from the assumption that targets are detected independently) is calculated from the data. Equations 1.1–1.3 show how to calculate the terms from the right part of Eq. 1. *P*(*A*) and *P*(*B*) are the hit rates for the “test” and “added” targets, respectively, on dual-target trials. Note, this requires two separate target classes (e.g., high- and low-salience targets) so that one can be defined as “test” and the other as “added”. *P*(*A* 2nd | *A*, *B*) is the proportion of dual-target trials with both targets detected in which the “test” target was detected after the “added” target.^2^

After computing this for each observer, if the left-hand side of Eq. 1 is not significantly different from the rightmost part, it would fail to reject the null hypothesis suggesting no SSM effect. If the left-hand side is significantly smaller than the right-hand side of the equation, then this difference would be the reported SSM effect. Alternatively, if the left-hand side is significantly larger than the right-hand side, there would be an anti-SSM effect.

Becker et al. (2020) demonstrated that by using this equation on the data reported in Adamo et al. (2019) (which is based on the same displays and targets used in many prior SSM studies; e.g., Adamo et al., 2013, 2017, 2018; Cain & Mitroff, 2013), this approach results in a smaller, but still significant, SSM effect compared to both the non-matched and matched search display design/data tested in Adamo et al. (2019) (see Becker et al., 2020 and Table 2 below for examples of how the SSM effect sizes change with different calculation methods).

**Table 2:** SSM Effect Calculated Four Different Ways from the Same Data Set

References	Different SSM error calculations on low-salience targets in Adamo et al. (2019) data	Results of t-test	Trials that are filtered
Fleck et al. (2010)	Single-low hit rate compared to low hit rate on dual-target trials where a high was detected *first or second*	*t* = 5.10 *p* < .001 Cohen’s *d* = 0.93	*Single:* None *Dual:* Remove trials where high-salience target was not found
Cain et al. (2013)	Single-low hit rate compared to low hit rate on dual-target trials where a high was detected *first*	*t* = 8.95 *p* < .001 Cohen’s *d* = 1.63	*Single:* None *Dual:* Remove trials where high-salience target was not found and trials where low-salience target was found first
Adamo et al. (2019)	*Matched* single-low compared to low hit rate on dual-target trials where a high was detected *first*	*t* = 8.22 *p* < .001 Cohen’s *d* = 1.50	*Single:* Remove trials that *do not match* the dual-target trials included in the analysis *Dual:* Remove trials where high-salience target was not found and trials where low-salience target was found first
Becker et al. (2020)	*Expected hit rate calculated from dual-target trials* compared to low hit rate on dual-target trials where a high was detected *first*	*t* = 5.21 *p* < .001 Cohen’s *d* = 0.95	*Single:* Remove all single-target trials *Dual:* Keep all dual-target trials to calculate *expected hit rate*. Remove trials where high-salience target was not found and trials where low-salience target was found first *to compare to expected hit rate*

The data analyzed were used in Adamo et al. (2019) in a multiple-target search with high- and low-salience targets (see Fig. 1). A two-tailed, paired-samples t-test was used to analyze the data. Fleck et al. (2010) originally calculated SSM errors by comparing the hit rate for single-target trials of one target type (e.g., low-salience ‘T’) to the hit rate of the same type of target when a different type of target was found on dual-target trials (e.g., low-salience ‘T’ when a high-salience ‘T’ was detected; See Fig. 1 graph). Cain and Mitroff (2013) later restricted to instances in which the target of interest was not found first on dual-target trials (e.g., hit rate for a low-salience ‘T’ when a high-salience ‘T’ was detected *first*). Adamo et al. (2019) then suggested changing the methods to be more in-line with radiological methods and use matching displays—displays where single- and dual-target trials were identical in target and distractor identity and location with the exception that one of the two targets were removed in the single-target trials (see Fig. 1 search displays). They suggested restricting the SSM calculation to only include matched single- and dual-target trials (e.g., filter the dual-target trials where the high-salience ‘T’ was found first and compare the hit rate for the “second” low-salience 'T' to the hit rate on its matched single, low-salience display). Becker et al. (2020) recommended using only dual-target trials to calculate the expected hit rate for a specific target (e.g., low-salience ‘T’; See Eq. 1). This value is compared to the same type of target when a different type of target was found first on dual-target trials (e.g., hit rate for low-salience targets on dual-target trials when a high-salience target was detected first)

## Future directions

### Attentional template theory

The collection of research related to SSM errors has suggested numerous explanations for why they occur. The three established theories (Satisfaction, Perceptual Set, and Resource Depletion) all received empirical support suggesting that there are temporal (e.g., Adamo et al., 2018; Stothart & Brockmole, 2019), perceptual (e.g., Biggs et al., 2015; Mitroff et al. 2015) and cognitive resource aspects to SSM errors (e.g., Adamo et al., 2013; Cain & Mitroff, 2013). These theories are often considered distinct without a unifying mechanism linking their separate predictions together. Given this, the current state of the field lacks the explanatory power necessary for a unified description of SSM errors. This hampers the ability to precisely determine when, where, and why SSM errors are likely to occur.

**Fig. 2:**
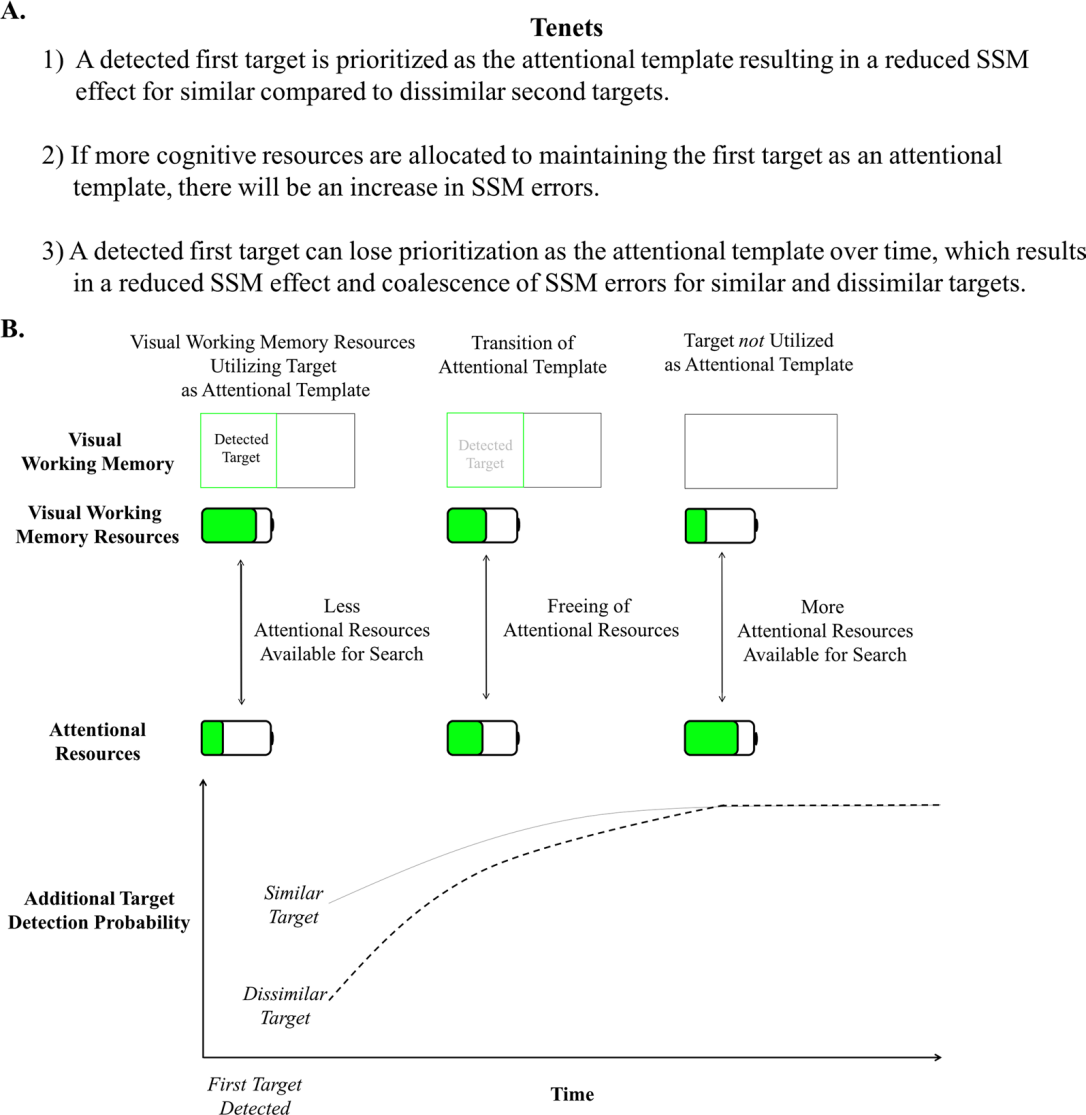
**A. Attentional Template Theory Tenets.** The tenets of the Attentional Template theory were motivated by the SSM error literature and the broader visual working memory, attention, and visual search literature (see Tenets 1, 2, and 3 subsections below). **B. Time Course of the Fluctuation of Working Memory and Attentional Resources.** The battery illustration depicts the capacity limitations of visual working memory and attention. The double-sided arrows between the batteries illustrate the flow of a shared cognitive resource that underlies both visual working memory and attention, and that when one cognitive process is prioritized, the remaining cognitive process is hindered. After first target detection, visual working memory resources are prioritized to maintain the first target as an attentional template. Consequently, there are fewer attentional resources available, which will overall decrease the probability of additional target detection. Detection for similar targets after detecting a first target will be better compared to dissimilar targets because a first target attentional template will prime attention towards similar targets. Over time the first target may lose its prioritization as the attentional template, which will free up visual working memory resulting in improved and unbiased detection for similar and dissimilar targets

Based on these shortcomings we propose a new Attentional Template theory (see Fig. 2), a novel mechanistic explanation for SSM errors, which ties together our current understanding of SSM errors and the attentional template literature. Our theory implements the attentional template as a mechanistic explanation for SSM errors so that future research can combine SSM error findings with ongoing visual search studies in cognitive science.

The attentional template is defined by cognitive scientists as an active representation of the search goal that can be held either within visual working memory (e.g., Gunseli et al., 2014; Schmidt & Zelinsky, 2017), or long term memory (e.g., Cunningham & Wolfe, 2014; Goldstein & Beck, 2018; Woodman et al., 2013), and attention is biased towards search items similar to the attentional template (e.g., Bravo & Farid, 2009; Vickery et al., 2005). To determine the impact that an attentional template has on search performance, observers are often asked to remember a target cue and then later search through a display where the target and/or distractors may be identical or similar to the cue. In general, search performance improves when a target matches or is more similar to the cue and is worse when a distractor matches the cue, suggesting that attention is guided by the cue's features (e.g., Bravo & Farid, 2009; Greene et al., 2015; Hout & Goldinger, 2015). However, search performance overall is worse when there are more visual working memory resources committed to maintaining an attentional template (Carlisle et al., 2011; Schmidt et al., 2014). This finding suggests that the cognitive resources (i.e., working memory and attentional resources) necessary for maintaining an attentional template in visual working memory and detecting a target are shared^3^ and that maintaining the target in visual working memory leaves less of the limited capacity system to process the search display. Therefore, this suggests a search benefit if the attentional template is offloaded from visual working memory to long term memory.

The Attentional Template theory predicts that additional targets are more likely to be missed because recognizing the first target results in re-activation of the attentional template through visual working memory (Luria & Vogel, 2011), which create a cascade of necessary target recognition processes that unfold over time (Luck et al., 1996; Vogel et al., 1998). The cognitive resources necessary for target recognition and establishing an item as the attentional template seem to be the same (e.g., Luck & Kappenman, 2012, Snyder & Foxe, 2010), and a target is processed within visual working memory before identification (e.g., Luria & Vogel, 2011; Tsubomi et al., 2013). Because of these findings, we hypothesize that detecting the first target results in that target becoming an attentional template that is maintained within visual working memory decreasing search performance for subsequent targets. This process results in SSM errors for subsequent targets because the newly formed attentional template is held active in visual working memory, which results in a search cost (e.g. Carlisle et al., 2011; Schmidt et al., 2014). This process taxes the cognitive resources required to recognize a new target as they are currently maintaining the first target an attentional template in visual working memory, which leads to an increased likelihood of missing a subsequent target. This is predicted because observers have a direct example of a possible target within view, which is automatically primed and reactivated in visual working memory. Given that the attentional template prior to search may be offloaded and maintained in long term memory, and thus not represented in visual working memory (e.g., Cunningham & Wolfe, 2014), this reactivation results in a search cost for subsequent targets. Consequently, the strong maintenance of the first target as an attentional in visual working memory, and the resulting cascade of target recognition processes, can negatively affect search and result in SSM errors. With this theory, it allows us to both provide a cohesive explanation for current SSM theories and findings, as well as offer novel predictions for the SSM error phenomena.

The prediction that a detected target may act as an attentional template for subsequent targets is also a hypothesis to account for a related phenomenon known as “runs” (Kristjánsson et al., 2014; Wolfe et al., 2019). “Runs,” the tendency to detect the same type of target as a previously detected target, are found when observers search for multiple targets of different types in a “foraging” visual search task. So rather than explaining why a target miss may occur, it explains why someone might detect several of the same types of targets in consecutive order. The existence of “runs” was demonstrated in visual search paradigms when observers foraged and searched for targets of multiple types (Kristjánsson et al., 2014; Wolfe et al., 2019). For example, Kristjánsson et al. (2014) found that observers would go on “runs” in a conjunction-based search task (where targets could share both the same color and shape as the distractor). Collectively these findings suggest that a detected target can act as an attentional template for subsequent targets. The Attentional Template theory takes this one step further and predicts that this is why SSM errors occur (i.e., SSM errors are the cost of maintaining the first target as an attentional template in visual working memory).

#### Tenet 1: a detected first target is prioritized as the attentional template resulting in a reduced SSM effect for similar vs. dissimilar second targets

This tenet of the Attentional Template theory can account for the perceptual, aspects of SSM errors observed within the SSM literature. In other words, it provides a mechanistic explanation for the Perceptual Set theory—observers are biased to search for targets perceptually similar to the attentional template and, consequently, less biased to search for dissimilar targets (e.g., Hout & Goldinger, 2015; Soto et al., 2005; Vickery et al., 2005). Because this finding is so consistent in the attentional template literature, it is the most straightforward, mechanistic explanation for why there is an increase in SSM errors when second targets are dissimilar to a detected first target (e.g., Biggs et al., 2015).

The SSM research suggests that observers are more like to find a second target when all the features match for both targets (i.e., the targets are identical), when the targets match on at least one feature (e.g., the targets are the same color), or when the targets are from the same category (e.g., the targets are guns in a carry-on bag; Biggs et al., 2015). It is currently unclear whether SSM errors will reduce more when the number of matching features increases (e.g., will there be fewer SSM errors when targets are the same rotation and color compared to when they are only the same color but different in rotation). Likewise, will SSM errors reduce when a feature is not an exact match but is more similar. For example, is a second target is more likely to be detected when it is a 10° difference in rotation compared to a 30° difference in rotation to a first target? Relatedly, if categorical similarity becomes more specific (e.g., animals vs. dogs vs. specific breeds of dogs), does second target detection improve?

Previous attentional template research suggests that when an attentional template and a target increase in similarity in a graded fashion across different feature dimensions (e.g., shape and orientation), search performance improves (Alexander et al., 2019; Bravo & Farid, 2009; Vickery et al., 2015). Search performance also improves when categorical specificity increases between an attentional template and a target (e.g., a boot vs. footwear; Maxfield & Zelinsky, 2012; Schmidt & Zelinsky, 2009). Based on these findings, the Attentional Template theory predicts that SSM errors will decrease as the similarity between the first and second targets increases in a graded fashion.

#### Tenet 2: if more cognitive resources are allocated to maintaining the first target as an attentional template, there will be an increase in SSM errors

This tenet can account for the cognitive resource aspects of SSM errors because it predicts that maintaining the first target as an attentional template is the reason cognitive resources are consumed by a first target (i.e., the prediction of the Resource Depletion theory). As described above, an attentional template can reside in visual working memory, which necessitates the utilization of cognitive resources to process the attentional template (e.g., Gunseli et al., 2014; Schmidt & Zelinsky, 2017) and produce the subsequent target recognition processes (Luck et al., 1996; Vogel et al., 1998). Consequently, maintaining an attentional template within visual working memory (rather than a longer-term memory store) can result in reduced visual search performance (Carlisle et al., 2011; Schmidt et al., 2014). A first target maintained as an attentional template in visual working memory could account for why SSM errors are attributed to an allocation of cognitive resources to a first target and result in fewer cognitive resources available to process a second target (e.g., Adamo et al., 2013, 2015, 2017; Cain & Mitroff, 2013; Stothart et al., 2017).

There is ample evidence within neuroscience to support the prediction that the allocation of cognitive resources^4^ to maintaining an attentional template can interfere with subsequent search performance. (1) Visual working memory and attention activate similar brain regions as measured by functional magnetic resonance imaging (e.g., Sheremata et al., 2018). (2) When there is reduced target-related visual working memory electroencephalogram (EEG) activity (i.e., lower contralateral delay activity; Vogel & Machizawa, 2004), subsequent search performance tends to improve (e.g., Carlisle et al., 2011; Schmidt et al., 2014). (3) The neural markers used in EEG to track an attentional template and recognize a target in a search display are the same (i.e., contralateral delay activity, P300, and alpha suppression; e.g., Fukuda et al. 2015; Gunseli, et al., 2014; Luck et al., 1996; Schmidt & Zelinski, 2017; Snyder & Foxe, 2010; Woodman et al., 2013).

Altogether, these findings suggest that the neural response to a first target can interfere with subsequent target detection (Luck et al., 1996; Vogel et al., 1998) and manipulations that utilize the same neural processes when recognizing an additional target should increase SSM errors. For example, the Attentional template theory would predict that a stronger contralateral delay activity and/or P300 response (neural markers both used to indicate the magnitude of working memory and attentional resources allocated to an attentional template/target) to a first target will correlate with a decrease in second target detection. Another prediction would be a reduced/absent P300 effect when observers fixate but miss a second target (similar to what has been observed with the attentional blink; Kok, 2001; Vogel et al., 1998). Finally, to assess how target similarity and cognitive resources are related (i.e., Tenets 1 and 2) the Attentional Template theory would predict that a greater P300 magnitude/duration in response to a first target would result in a longer duration of improved similar target detection compared to dissimilar target detection.

#### Tenet 3: a detected first target can lose prioritization as the attentional template over time

Concerning the temporal aspects of SSM errors (i.e., the evidence for the Satisfaction theory), the Attentional Template theory predicts that observers will be less affected by a first target attentional template when more time is spent searching after first target detection. In other words, observers are initially biased to search for similar targets after a first target is found. However, as time passes, the first target may no longer be held as an attentional template in visual working memory because the target recognition process has completed, allowing for more efficient processing in search.

Evidence suggests that search performance may improve when an attentional template is transferred out of visual working memory to activated long term memory (e.g., Carlisle et al., 2011; Goldstein & Beck, 2018; Schmidt et al., 2014; Woodman et al., 2013). This is due to visual working memory’s involvement in perceptual processing and visual search (e.g., Cunningham & Wolfe, 2014; Drew & Vogel, 2008; Luria & Vogel, 2011; Tsubomi et al., 2013) and removal of the attentional template from visual working memory can allow for more efficient processing of search items (e.g., Schmidt & Zelinsky, 2014).

If the attentional template is more likely to be removed from visual working memory overtime, this can explain why observers are less likely to make an SSM error when they search for longer after finding a first target (Adamo et al., 2018; Stothart & Brockmole, 2019).^5^ Furthermore, it can explain why there is an attentional blink-like pattern in SSM errors (Adamo et al., 2013) and why there is a relationship between the width of an individuals' attentional blink and the likelihood of making an SSM error (Adamo et al., 2017). The maintenance of a first target as an attentional template initially interferes with search performance during the blink window and observers who maintain the first target for longer should be more susceptible to SSM errors.

While SSM errors overall have been shown to improve the longer observers search after finding a first target (Adamo et al., 2018; Stothart & Brockmole, 2019), a novel way to test this prediction is to investigate whether SSM rates for similar targets and dissimilar targets coalesce the longer observers search (See Fig. 2B). If the first target is initially utilized as an attentional template, the Attentional Template theory would predict that observers will be biased to search for similar targets compared to dissimilar targets. However, as time passes, it would predict that dissimilar target detection would increase to the rate of similar target detection as fewer resources are allocated to preserving the first target as the attentional template.

#### Final thoughts on the attentional template theory and SSM errors

While the Attentional Template theory is largely untested, it offers the attentional template as an established mechanism within the cognitive science literature that can be tested in future SSM research. It integrates SSM findings from the broader attention, visual working memory, and visual search literature. All previous SSM error theories were initially proposed within radiology (Berbaum et al., 1990, 1991; Tuddenham, 1962) and did not account for more recent findings from research in cognitive science. Given that no prior SSM theory provided a mechanistic explanation for SSM errors, hopefully, future SSM research can use the attentional template as a foundation to investigate SSM errors. For example, there is a rich literature in cognitive science investigating the attentional template within neuroscience (e.g., see Olivers et al., 2011; Vries et al., 2017; Zelinsky & Bisley, 2015), which may now be applied to the SSM literature. Overall the predictions of the Attentional Template theory should lead to a better theoretical understanding of SSM errors and offer many new avenues of research to determine why the detection of one target impacts another target.

### Moving towards a standardized assessment of SSM errors

As described previously, there have been many different methods and analyses proposed to study SSM errors across radiology and cognitive science (e.g., Adamo et al., 2019; Becker et al., 2020; Berbaum et al., 1990; Cain & Mitroff, 2013; Stothart & Brockmole, 2019). While radiology has primarily used matched single- and dual-abnormality images to determine the impact an “added” abnormality has on a “test” abnormality, it does not restrict the SSM error analyses to situations in which the “added” abnormality was detected first. Unfortunately, this style of analysis makes it difficult to assess the significance of initial target detection upon the detection of subsequent targets, which all SSM theories are based on.

Cognitive science has largely restricted its SSM analyses to second-target hit rate relative to single-target hit rate to determine the impact on successive target detection. While reliance on heuristically derived metrics has led to an overestimation of the magnitude of the SSM effect in certain cases (see Adamo et al, 2019; Becker et al., 2020), calculations have been developed to test current SSM theories. To calculate the unbiased estimation of SSM effect sizes, researchers could use Eq. 1 (Becker et al., 2020) as it is theoretically well-founded. When applying this method, researchers should consider estimating the effect that detecting a "test" target has on the subsequent detection of an "added" target (i.e., *P*(*B* | *A* 1st)) to identify possible target identity interactions. However, this equation relies on using multiple target types (e.g. high- and low-salience targets or two different classes of targets), measuring binary decisions, and seeking to evaluate against the null hypothesis that targets are detected independently.

Future cognitive science studies may also consider incorporating signal detection measures as radiologists have to account for false alarm rates and confidence ratings in their SSM calculations. For example, if SSM errors are driven by observers searching more conservatively (i.e., a decrease in hit rate and false alarms), this should be accounted for in future SSM error calculations. This is especially important given that radiologists are concerned with making a false alarm, as it can lead to an unnecessary biopsy and potential patient harm. Thus, non-hit rate measures should be compared to current SSM calculations in cognitive science and potentially incorporated as indicators of search performance if cognitive studies are to be more applicable to radiologists. Ultimately, a more standardized calculation will greatly benefit our understanding of SSM errors across radiology and cognitive science.

### Non-subsequent search misses?

While not explicitly discussed within the SSM literature, future work across radiology and cognitive science may also want to consider another way that a target may impact the detection of an additional target, which we deem the “mere presence effect”. These are potential situations when the “added” target in the display reduces the hit rate for the target of interest even if the “added” target is not detected first. Although “mere presence effect” errors are clearly different from SSM errors, they could contribute to some SSM metrics that do not appropriately differentiate between these two important sources of error. Future research will need to appropriately isolate misses attributable to this “mere presence effect” from those attributable to the SSM effect alone (i.e., those identified using Eq. 1; see “Appendix” for further discussion of a potential way to calculate the mere presence effect).

### New avenues of SSM error research

The fields of radiology and cognitive science have investigated why, when, and where SSM errors occur, yet their research has largely been independent of one another. A great first step in advancing our understanding of SSM errors would be to have a cyclical approach to studying SSM errors between radiology and cognitive science. On one hand, radiology has focused on identifying the pervasiveness of the SSM errors across different radiological subfields (e.g., chest, skeletal, abdominal), theorizing the cause of SSM errors, and whether different target detection tools alleviate SSM errors (e.g., Berbaum et al., 1990; Samuel et al., 1995). On the other hand, research in cognitive science has generally focused on testing the different SSM error theories and theorizing how their findings will translate to real-world contexts (e.g., Adamo et al., 2018; Biggs et al., 2015; Cain & Mitroff, 2013; Stothart & Brockmole, 2019). Thus, there is a critical need to relate cognitive science findings to radiology and determine how the results observed within simplified-search displays with novice observers translates to medical images with radiologist as observers.

Concerning the temporal component of SSM errors, the time spent searching after finding the first target, a measure found to correlate with SSM errors in simplified search displays (Adamo et al., 2018; Stothart & Brockmole, 2019), could be investigated to determine if radiologists are less likely to make an SSM error when they search for longer after finding the first abnormality. For the perceptual aspect of SSM errors, researchers could investigate whether radiologists are more likely to miss one type of abnormality (e.g., a nodule within a chest image) when a different abnormality is detected first (e.g., a broken rib) compared to a similar abnormality (e.g., another nodule). For the resource aspect of SSM errors, radiologists could investigate the time frame of the attentional blink (~ 200–500 ms) to determine if radiologists are more likely to miss a second-abnormality when it is fixated in that time window after a first abnormality or how other attentional taxing influences (e.g., clutter) impacts SSM error rates, similar to how they are impacted within simplified search displays (e.g., Adamo et al., 2013, 2017).

Another factor that may be contributing to real-world SSM errors is the frequency rates of radiological abnormalities. While low target prevalence (i.e., observers rarely see any targets) has been demonstrated to increase SSM errors (Chen & Rich, 2019), it is unclear if low target frequency (i.e., observers rarely see a specific target) affects SSM errors in the same way. It is important to determine whether SSM errors are mitigated by frequency given that abnormalities appear at different frequencies. For example, the hit rate for incidental findings, (i.e., an abnormality that the observer is not specifically searching for) can be impacted by target frequency. Wolfe et al. (2017; Experiment 2) demonstrated that when observers are searching for specific and categorically defined targets, an incidental finding from a rare category of possible targets is more likely to be missed compared to an incidental finding from a common category of possible targets. Extrapolating these findings to SSM errors, if an observer is specifically looking for and finds one type of abnormality (e.g., a spiculated mass in a mammogram), how will abnormality frequency impact the detection of an additional abnormality of a different category (e.g., a non-specific density)? When studying SSM errors within chest radiographs, there can be upwards of 22 different types of native abnormalities used to test whether an added nodule affects the detection of the native abnormality (Berbaum et al., 2015; Krupinski et al., 2017). Given that nodules are the most commonly occurring type of abnormality in the images tested, if one type of abnormality is more frequent within a medical image (e.g., pneumonia) appears with the nodule, it may be more prone to SSM errors compared to less frequent abnormalities (e.g., lytic lesion of the right scapula).

Beyond learning the causes of and influences on SSM errors, research needs to use this information to better develop ways of mitigating SSM errors. For example, motivation, training, and advances in imaging (i.e., moving from 2 to 3D search images) are ripe areas of research that need to be investigated to determine whether any of them can reduce/mitigate SSM errors. In regards to improvements in imaging technology, radiological fields such as breast cancer detection are moving from 2D mammography to 3D tomosynthesis. Mammography has been the tool of choice for quite some time when searching for breast cancer (Gandomkar & Mello-Thoms, 2019), but radiologists can miss up to 30% of cancers with mammography (Ekpo et al., 2018). To improve detection and reduce recalls, radiologists have turned to tomosynthesis. Tomosynthesis allows for a pseudo-3D search where the breast volume is divided into many 2D slices and allows radiologists to scroll through slices and search in-depth. Radiologists detect more cancers (Ciatto et al, 2013) and have fewer recalls with tomosynthesis compared to mammography (Bernardi et al., 2012). However, it is unknown whether tomosynthesis/searching in 3D reduces SSM errors. This is a fruitful area of future research because these types of 3D searches may be applied to other fields of radiology or even other critical searches such as those conducted by baggage screeners.

## Conclusions

Unlike other attentional phenomena (e.g., the attentional blink; Broadbent & Broadbent, 1987) SSM errors were not discovered in a lab but were instead discovered because abnormalities were missed in radiological images (Smith, 1967; Tuddenham, 1962). Beyond radiology, SSM errors have been shown to occur in many different types of search images, including airport baggage screening simulations (e.g., Biggs et al., 2015; Mitroff et al., 2015), diagnostic medicine (Kuhn, 2002), cytology (Bowditch, 1996), and driving simulations (Sall & Feng, 2019). Researchers aim to evaluate and quantify SSM errors in the laboratory to reduce the potential harm caused by professionals missing lifesaving targets in visual searches. Mechanistic explanations should be pursued and rigorously tested as that may allow us to mitigate SSM errors in the future. While the methodologies and analyses used to estimate SSM errors have been evolving, convergence should encourage collaboration between the fields for the mutual benefit of preventing abnormalities/target misses. If research in both radiology and cognitive science can be combined with the common goal of mitigating search misses, then missed diagnoses can be reduced and lives potentially saved.

Hopefully, this review has demonstrated that SSM errors are not merely due to the long-held notion that observers become “satisfied” with the meaning of the image and prematurely terminate their search, as was originally predicted (Tuddenham, 1962). Rather, SSM errors occur because our minds maintain and attend to items in the visual world in specific ways, which have beneficial and detrimental consequences in visual search.

## Data Availability

Not applicable.

## Appendix: Isolating the “mere presence effect”

While not explicitly discussed within the SSM literature the mere presence of an “added” target (regardless if it is detected) may contribute to missing a “test” target. For example, radiologists are known to determine with above-chance accuracy that an abnormality is present when a medical image is flashed as quickly as 200 ms (e.g., Kundel & Nodine, 1975). This finding suggests that determining whether an abnormality is present may not necessarily occur at detection, but can be inferred from a global response that establishes content at the beginning of the search. Furthermore, radiologist can detect an abnormality, but not recognize it as an abnormality (i.e., a decision making error; Nodine & Kundel, 1987), which may affect identification of future abnormalities.

As suggested in the main text, using Eq. 1 is appropriate for calculating the SSM effect. However, the best way to isolate any potential mere presence effect remains to be determined as it has not yet been discussed within the SSM literature (as this was realized in the writing of this manuscript). One could consider testing for a “mere presence effect” by comparing overall “test” target (i.e., a target potentially affected by the mere presence effect) hit rates on dual- to single-target trials using the equation:

2 $$P\left( A \right)\, {:=}\, \frac{{{\text{Single trials}}\;w/A\;{\text{detected}}}}{{{\text{Single trials}}\;w/A}}\frac{ < }{ > }\frac{{{\text{Dual trials}}\;w/A\;{\text{detected}}}}{{{\text{Dual trials}}\;w/A}}$$

This comparison could be used to identify a mere presence effect in the absence of an SSM effect. This distinction is addressing a slightly different question, rather than assessing how the detection of a target affects the detection of a second target, the "mere” presence calculation includes the effects of undetected targets as well. Importantly, the overall “test” target hit rate on dual-target trials is made up of trials when the “test” target is detected either first or second. Thus, if there is an SSM effect, the overall “test” target hit rate could be lower on dual-target trials, even in the absence of a “mere presence effect” (i.e., the overall increase in misses could be fully explained by second-target misses). Thus, in addition to reporting comparisons of expected second-target detection probabilities, as discussed previously, researchers could consider comparisons based on the expected probability of detecting the “test” target first:

3 $$P\left( {A\;1{\text{st}}} \right)\, {:=}\, \frac{{{\text{Dual trials}}\;w/A\;{\text{detected}}\;1{\text{st}}}}{{\text{Dual trials}}}\frac{ < }{ > }P\left( A \right)\left( {1 - P\left( B \right)} \right) + P\left( A \right)P\left( B \right)P(B\;2{\text{nd}}|A,B)$$

3.1 $$P(B\;2{\text{nd}}|A,B)\, {:=}\, \frac{{{\text{Dual trials}}\;w/A\;{\text{detected}}\;{\mathbf{then}}\;B\;{\text{detected}}}}{{{\text{Dual trials}}\;w/A\;{\text{detected}}\;{\mathbf{and}}\;B\;{\text{detected}}}}$$

where each of the overall “test” and “added” hit rates on the right of Eq. 3 (i.e., *P*(*A*) and *P*(*B*), respectively) can be computed from single-target trials. *P*(*B* 2nd | *A*, *B*) is the proportion of dual-target trials with both targets detected in which the “added target” is detected second (Eq. 3.1). If the left-hand side of Eq. 3 is significantly different from (either less than or greater than) the right-hand side, then there may be a mere presence effect (either a suppression or a boost depending on the sign of the difference).

## Footnotes

1 This was the first paper that used the term “Subsequent Search Misses” instead of “Satisfaction of Search.” Since “Satisfaction of Search” implied that misses occurred because of “satisfaction,” and there was no conclusive evidence for the “Satisfaction” theory at the time, the name was changed to avoid confusion. The acronym "SSM" also uses the same letters as the first letters of the authors' first names (i.e., an SSM error "Easter Egg").

2 Jeremy Wolfe proposed a similar approach to calculating SSM errors based solely on detection probabilities of an "added" and "test" target in Biggs (2017; See Eq. 4). In this equation, the hit rate for an added and test target for single-target trials are multiplied together and established as the baseline and compared to the hit rate for the “test” target on dual-target trials (to determine whether there is an SSM effect). However, this method for calculating SSM errors cannot directly assess the impact that one target has on another like the equation in Becker et al. (2020) because it cannot differentiate whether the impact of a first target is due to “subsequence” (detecting a target second) or “mere” exposure (See “Appendix”).

3 Many findings and theories suggest that visual working memory acts as internally orientated attention, similar to how the spatial focus of attention is allocated to external stimuli (e.g., Awh & Jonides, 2001; Drew et al., 2016; Kiyonaga & Egner, 2013). Recent neuroimaging evidence suggests that visual working memory and attention activate similar brain regions (e.g., Sheremata et al., 2018) and that when multiple objects appear on the screen, they compete for attention and working memory resources (e.g., Desimone & Duncan, 1995; Fiebelkorn et al., 2013).

4 Whereas the idea of “cognitive resources” is often used to explain behavioral data, (e.g., Adamo et al., 2013), neural evidence suggests that a more physiologically accurate way to conceptualize “resources” may be a neural process that unfolds over time. In other words, SSM errors may occur because the neural mechanisms utilized to process and maintain the first target as an attentional template in visual working memory are the same neural mechanisms needed to detect a second target. Because the processing of the first target has not yet concluded, the second target is missed.

5 Another plausible explanation for why SSM errors improve over time may be simply because the longer observers spend searching, the more likely they are to find an additional target. Thank you to Todd Horowitz for pointing this possibility out to us.
